# MiR-203a-3p regulates TGF-β1-induced epithelial–mesenchymal transition (EMT) in asthma by regulating Smad3 pathway through SIX1

**DOI:** 10.1042/BSR20192645

**Published:** 2020-02-28

**Authors:** Qi Fan, Yu Jian

**Affiliations:** Department of Emergency Medicine, Jingzhou Central Hospital, Jingzhou, Hubei, China

**Keywords:** asthma, EMT, miR-203a-3p, SIX1, Smad3, TGF-β1

## Abstract

Asthma is a common chronic airway disease with increasing prevalence. MicroRNAs act as vital regulators in cell progressions and have been identified to play crucial roles in asthma. The objective of the present study is to clarify the molecular mechanism of miR-203a-3p in the development of asthma. The expression of miR-203a-3p and Sine oculis homeobox homolog 1 (SIX1) were detected by quantitative real-time polymerase chain reaction (qRT-PCR). The protein levels of SIX1, fibronectin, E-cadherin, vimentin, phosphorylated-drosophila mothers against decapentaplegic 3 (p-Smad3) and Smad3 were measured by Western blot. The interaction between miR-203a-3p and SIX1 was confirmed by dual-luciferase reporter assay and RNA immunoprecipitation (RIP) assay. MiR-203a-3p was down-regulated and SIX1 was up-regulated in asthma serums, respectively. Transforming growth factor-β1 (TGF-β1) treatment induced the reduction of miR-203a-3p and the enhancement of SIX1 in BEAS-2B and 16HBE cells in a time-dependent manner. Subsequently, functional experiments showed the promotion of epithelial–mesenchymal transition (EMT) induced by TGF-β1 treatment could be reversed by miR-203a-3p re-expression or SIX1 deletion in BEAS-2B and 16HBE cells. SIX1 was identified as a target of miR-203a-3p and negatively regulated by miR-203a-3p. Then rescue assay indicated that overexpressed miR-203a-3p ameliorated TGF-β1 induced EMT by regulating SIX1 in BEAS-2B and 16HBE cells. Moreover, miR-203a-3p/SIX1 axis regulated TGF-β1 mediated EMT process in bronchial epithelial cells through phosphorylating Smad3. These results demonstrated that MiR-203a-3p modulated TGF-β1-induced EMT in asthma by regulating Smad3 pathway through targeting SIX1.

## Introduction

Asthma, as a common chronic airway disease, affects approximately 300 million people in the world [1]. Up to now, asthma is still a major problem to public health, and the prevalence of asthma is increasing in developing countries and undeveloped countries because of late diagnosis and poor treatment [2]. Although asthma is commonly diagnosed in childhood, it occurs in all age groups [3]. Ra et al. reported that there was no significant difference between the prevalence rate of the elderly (>65) and that of the young people (18–34) [4]. A lot of stimuli, including cold air and exercise, and other risk factors such as smoking, obesity and rhinitis contribute to asthma incidence [5–7]. In general, asthma patients often represent chronic airway inflammation and remodeling reactions [8]. However, up to now there are no feasible methods to restore airway remodeling. Currently, the main treatment for asthma is inhaling corticosteroids. However, a considerable number of patients are still incurable despite inhalation of high concentrations of corticosteroids [9]. Thus, a better understanding of the molecular mechanism in asthma is essential for the treatment and prevention of asthma.

MicroRNAs (miRNAs), a class of endogenous non-coding RNA with the length of 18–23 nucleotides, have been identified to involve in many biological processes [10]. In recent years, plenty of miRNA were proved to associate with cancer cell development and progression [11]. MiR-203a-3p is a functional miRNA, and has been identified to involve in the development of many cancers. For example, miR-203a-3p acted as an important tumor suppressor to affect gastric cancer cell proliferation by targeting IGF-1R [12]. Also, Chen et al. demonstrated that miR-203a-3p was a novel molecular therapeutic target of colorectal cancer, which has been proved to enhance colorectal cancer proliferation and migration [13]. Besides that, bio-informatics analysis revealed that the expression of miR-203a-3p was decreased evidently in the bronchial epithelial cells, indicating that miR-203a-3p might be the potential target for the treatment of asthma [14]. However, the function and molecular mechanism of miR-203a-3p in the development of asthma need further study.

Mature miRNA regulated target mRNA expression by binding the 3′-untranslated region (3′-UTR) with RNA-induced silencing complex (RISC). Whether there is a potential relationship between miR-203a-3p and Sine oculis homeobox homolog 1 (SIX1) has not been reported. SIX1 is a member of sine oculis homeobox gene family, and has been revealed to play important regulatory roles in many tissues and organs [15,16]. Besides, SIX1 also was reported to involve in tumorigenesis of many human cancers. For instance, previous report demonstrated that SIX1 expression was markedly increased in NSCLC and miR-204/SIX1 pathway might be a therapeutic approach for the treatment of lung cancer [17]. SIX1 might be a novel potential target for hepatocellular cancer (HCC) treatment through participating in the regulation of cell proliferation in HCC [18]. Zeng et al. reported that SIX1 enhanced epithelial–mesenchymal transition (EMT) in breast cancer by interaction with miR-204-5p [19]. Additionally, recent study has indicated that SIX1 was remarkably elevated in asthmatic mice and played a major role in transforming growth factor-β (TGF-β1)-induced EMT and pulmonary fibrosis in asthma [20]. It has been reported that dysregulation of the TGF-β1/Smad pathway is necessary for bronchial epithelial cell proliferation in asthma patients. TGF-β1 can initiate cellular response, inducing phosphorylation of Smad2/Smad3 [21]. In addition, miRNAs have been found to implicate in the regulation of TGF-β1/Smad pathway. For instance, miR-485 overexpression suppressed cell proliferation and induced apoptosis of airway smooth muscle cells (ASMCs) through the Smurf2-mediated TGF-β/Smads signaling pathway in mice with chronic asthma [22]. MiR-744 repressed bronchial epithelial cell proliferation by regulating Smad3 via targeting TGF-β1 in severe asthma [21].

In the present study, we investigated the expression and the roles of miR-203a-3p and SIX1 in asthma, and elucidated the regulatory relationship among miR-203a-3p, SIX1 and Smad3 in the development of asthma.

## Materials and methods

### Serum samples

A total of 50 serum samples (25 serum from asthma patients and 25 from healthy people) were obtained from Department of Emergency Medicine, Jingzhou Central Hospital and immediately were stored at −80°C. All patients were signed the written informed consents prior to research. Besides, all the methods in our study were authorized by the Ethics Committee of Department of Emergency Medicine, Jingzhou Central Hospital. Clinical characteristics of all subjects are summarized in Table 1.

**Table 1:** Clinical characteristics of asthma patients

Clinical feature	Asthma cases (25)	Healthy controls(25)
Age (years)	43 ± 8	46 ± 7
Sex (male/female)	14/11	12/13
FEV1 (%)	67.4 ± 7.6	98.4 ± 8.7*
FEV1/FVC ratio (%)	68.3 ± 8.5	80.1 ± 6.7
Serum IgE (IU/ml)	138.3 ± 10.6	33.5 ± 9.5*

Healthy controls versus asthma cases, *P < 0.05.

### Cell culture and treatment of TGF-β1

Human bronchial epithelial (HBE) cell lines BEAS-2B were purchased from American Tissue Culture Collection (ATCC, Manassas, VA, U.S.A) and 16HBE was achieved from Cancer Research Institute of Beijing, China. All cells were maintained in Dulbecco’s Modified Eagle Medium (DMEM; Gibco, Carlsbad, CA, U.S.A.) supplemented with 10% fetal bovine serum (FBS; Thermo Fisher Scientific, Waltham, MA, U.S.A.) at 37°C with 5% CO_2_. TGF-β1 was purchased from Selleck (Selleck, Shanghai, China). The TGF-β1 was dissolved in 10 mM citric acid and stored at −20°C. BEAS-2B and 16HBE cells were treated with 10 ng/ml TGF-β1 for 0, 12, 24 and 48 h, respectively. Cells were harvested for further study.

### Quantitative real-time PCR

Total RNA was extracted using TRIzol reagent (Invitrogen, Carlsbad, CA, U.S.A.) from all serum samples and bronchial epithelial cells. The quality and quantity of RNA were determined by Nanodrop 2000 (Thermo Fisher Scientific). The first strand of cDNA was synthesized by SuperScript III reverse transcriptase kit (Thermo Fisher Scientific) referring to the user manual. QRT-PCR was performed by TransStart Top Green qPCR SuperMix (TransGen, Beijing, China) with the procedure: 94°C 3 min, 40 cycles of 94°C 10 s and 60°C 10 s. Glyceraldehyde-3-phosphate dehydrogenase (GAPDH) and U6 were regarded as the inner control. All the qRT-PCR data were standardized with 2^−ΔΔCt^ method. The primers of GAPDH and special primer of miR-203a-3p were collected from Songon Biotech (Songon Biotech, Shanghai, China) and listed in the Table 2. All experiments were repeated for three times.

**Table 2:** Special primer sequences for qRT-PCR

Gene	Forward	Reverse
miR-203a-3p	CCGGTGAAATGTTTAGGACCACTAG	GCCGCGTGAAATGTTTAGG
SIX1	CGCGCACAATCCCTACCCATCGCC	CTTCCAGAGGAGAGAGTTGGTTCTG
GAPDH	GACTCCACTCACGGCAAATTCA	TCGCTCCTGGAAGATGGTGAT
U6	GTAGATACTGCAGTACG	ATCGCATGACGTACCTGAGC

### Western blot assay

The protein was isolated using RIPA lysis buffer (Beyotime, Shanghai, China) and incubated with protease inhibitors (Thermo Fisher Scientific). Then, the amount of protein was measured by bicinchoninic acid (BCA) protein assay kit (Beyotime). Next, protein was subjected to sodium dodecyl sulfate-polyacrylamide gel electrophoresis (SDS-PAGE) gel. Subsequently, the protein in the gel were transferred onto polyvinylidene fluoride (PVDF) membranes (Millipore, Billerica, MA, U.S.A.) and blocked with 5% nonfat milk. The primary antibodies against SIX1, GAPDH, fibronectin, E-cadherin, vimentin, Smad3 and p-Smad3 were purchased from Abcam (Abcam, Cambridge, MA, U.S.A.) and incubated the membranes overnight at 4°C. After being washed with phosphate-buffered solution containing Tween-20 (PBST) for 5–6 times, the membranes were interacted with HRP-conjugated secondary antibody (Abcam). Finally, the protein on membranes was visualized by using Pierce™ ECL Western blotting Substrate (Thermo Fisher Scientific) and quantitated by Image Lab software (Bio-Rad, Hercules, CA, U.S.A.).

### Dual-luciferase reporter assay

The potential binding sites between miR-203a-3p and SIX1 were predicted using TargetScan online tool. To confirm the interaction between miR-203a-3p and SIX1, 3′-UTR of SIX1 wild-type (wt) and mutant (mut) were amplified and sub-cloned into the luciferase reporter vectors (Promega, Madison, WI, U.S.A.). All luciferase vectors were constructed by Hanbio Biotechnology Co., Ltd and co-transfected into BEAS-2B and 16HBE cells with NC or miR-203a-3p. After transfection for 48 h, the luciferase activities were detected by dual-luciferase assay kit (Promega) according to instructions.

### RNA immunoprecipitation (RIP) assay

The RIP assay was conducted using magna RIP RNA-Binding Protein Immunoprecipitation Kit (Millipore). BEAS-2b and 16HBE cells were transfected with NC and miR-203a-3p. After two-day transfection, cells were harvested and lysed in 100 μl RIP Lysis Buffer. The protease inhibitor cocktail and RNase inhibitors were needed in the process of dissolution. Next, Ago2 or IgG antibodies (Millipore) were added into cell lysates and incubated overnight at 4°C. Finally, the immunoprecipitated RNA was obtained by the RNeasy MinElute Cleanup Kit (Qiagen, Duesseldorf, Germany), and the abundance of RNA was measured by quantitative real-time PCR.

### Statistical analysis

Statistical analyses were determined by SPSS software (IBM, Armonk, NY, U.S.A.). All data from three independent replicates were displayed as the mean ± standard deviation. Statistically differences were calculated by Student’s *t* test or one-way analysis of variance (ANOVA) followed by Tukey’s post hoc test. The correlation analysis was performed using Pearson analysis. The *P* value less than 0.05 was considered as statistically significant.

## Results

### MiR-203a-3p was down-regulated and SIX1 was up-regulated in Asthma serum samples

**Figure 1:**
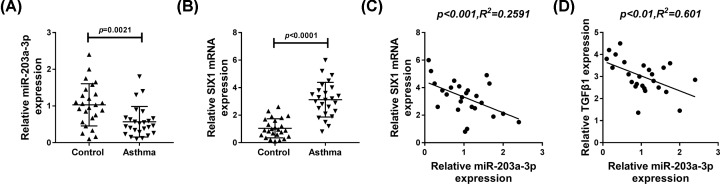
The expression of miR-203a-3p and SIX1 in asthma serums (**A** and **B**) The levels of miR-203a-3p (*P* = 0.0021) and SIX1 mRNA (*P* < 0.0001) were detected by qRT-PCR in asthma and control groups. (**C**) The correlation analysis between miR-203a-3p and SIX1 was determined by Pearson analysis. (**D**) The correlation analysis between miR-203a-3p and TGF-β1 was analyzed by Pearson analysis.

To explore the potential roles of miR-203a-3p and SIX1 in asthma development, their expression in 25 asthma serums and 25 normal serums was detected. Compared with the control group, the level of miR-203a-3p was significantly decreased in asthma serum samples (*P* = 0.0021; Figure 1A), while SIX1 expression was markedly increased in asthma serum samples (*P* < 0.0001; Figure 1B). Notably, a negative correlation between miR-203a-3p and SIX1 in asthma serum samples was observed (*P* < 0.001, *R*^2^ = 0.2591; Figure 1C). Moreover, we also found miR-203a-3p was negatively correlated with TGF-β1 in asthma serum samples (*P* < 0.001, *R*^2^ = 0.601; Figure 1D), suggesting the possible effects of miR-203a-3p in asthma EMT.

### TGF-β1 suppressed miR-203-3p expression and promoted SIX1 expression in BEAS-2B and 16HBE cells

**Figure 2:**
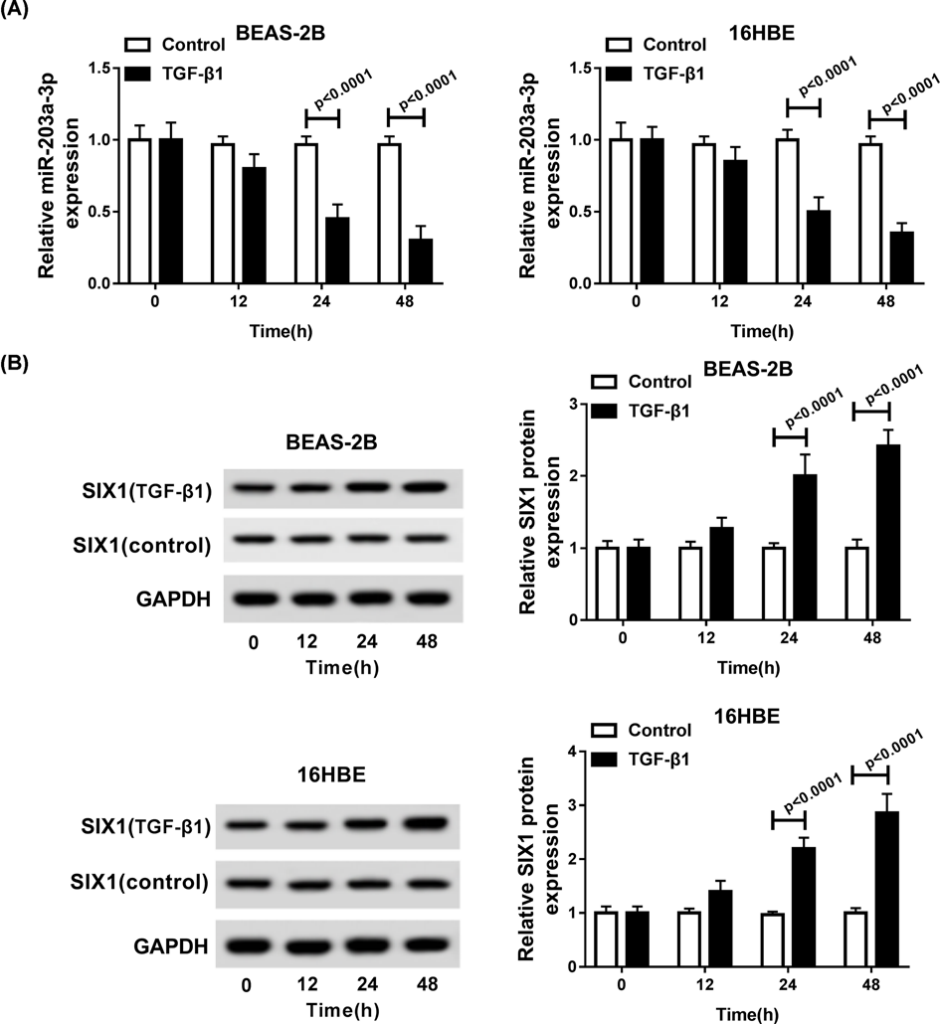
The expression of miR-203a-3p and SIX1 in TGF-β1-induced BEAS-2B and 16HBE cells (**A**) The expression of miR-203a-3p was measured by qRT-PCR in BEAS-2B and 16HBE cells after treatment with TGF-β1 for 0, 12, 24 and 48 h. (**B**) The protein level of SIX1 was determined by Western blot in TGF-β1-induced BEAS-2B and 16HBE cells.

To explore the biological effects of TGF-β1 in asthma *in vitro*. First, the levels of miR-203-3p and SIX1 were detected. The qRT-PCR results showed TGF-β1 treatment induced significant reduction of miR-203a-3p levels in BEAS-2B and 16HBE cells in a time-dependent manner (Figure 2A). Meanwhile, Western blot analysis determined that SIX1 level was significantly enhanced by TGF-β1 treatment in BEAS-2B and 16HBE cells in a time-dependent manner (Figure 2B). In a word, TGF-β1 could regulate miR-203-3p and SIX1 expression *in vitro*.

### MiR-203a-3p overexpression inhibited epithelial–mesenchymal transition of TGF-β1-induced bronchial epithelial cells

**Figure 3:**
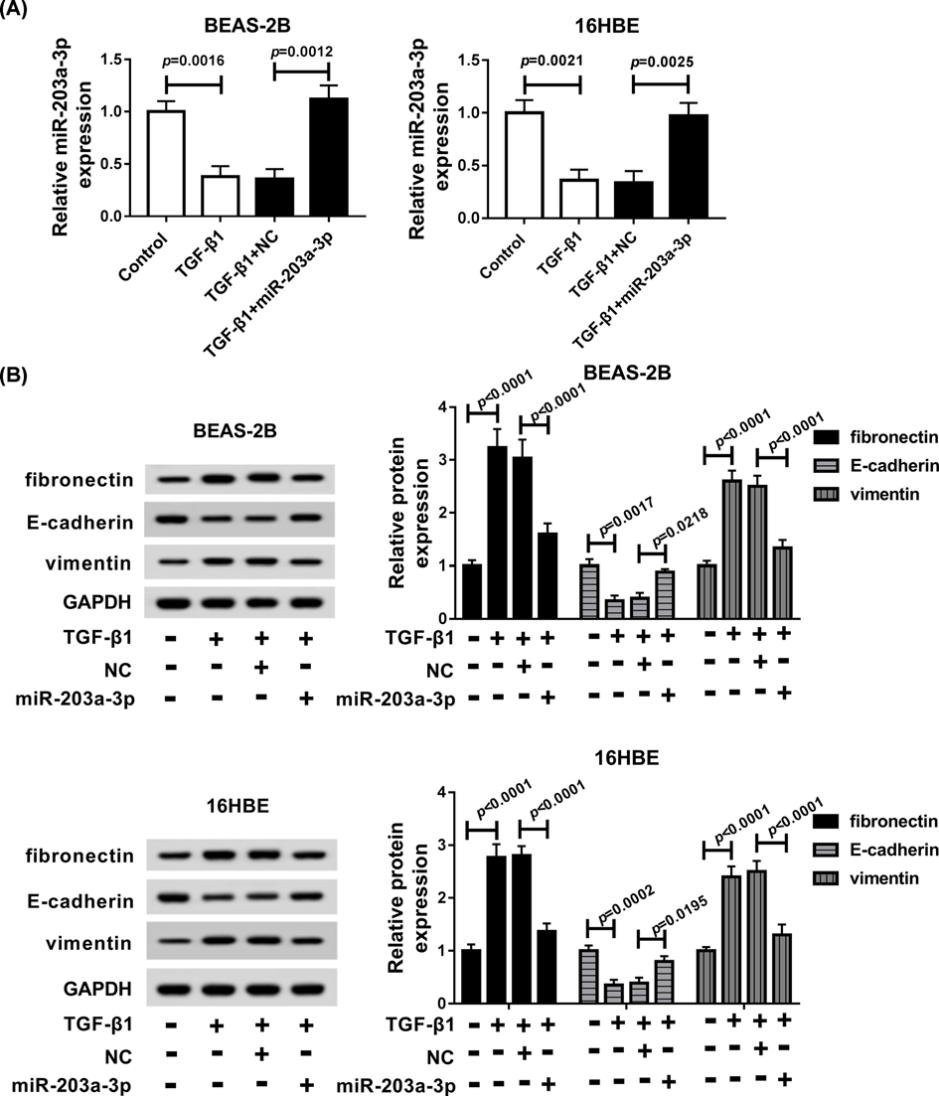
MiR-203a-3p overexpression suppressed TGF-β1-induced EMT in BEAS-2B and 16HBE cells BEAS-2B and 16HBE cells were treated with TGF-β1, TGF-β1 + NC, TGF-β1 + miR-203a-3p. (**A**) The expression of miR-203a-3p was detected by qRT-PCR in BEAS-2B and 16HBE cells. (**B**) The levels of proteins (fibronectin, E-cadherin, vimentin and GAPDH) were detected by Western blot assay in BEAS-2B and 16HBE cells.

To investigate the role of miR-203a-3p on epithelial–mesenchymal transition (EMT) in asthma, NC or miR-203a-3p was transfected into TGF-β1-induced BEAS-2B and 16HBE cells, respectively. As expected, miR-203a-3p level was decreased by TGF-β1 treatment (*P* = 0.0016 and *P* = 0.0021), but was dramatically elevated by following miR-203a-3p mimics transfection in BEAS-2B (*P* = 0.0012) and 16HBE (*P* = 0.0025) cells (Figure 3A). Moreover, we also found miR-203a-3p was increased in BEAS-2B and 16HBE cells transfected with miR-203a-3p mimics without TGF-β1 treatment (*P* = 0.0002; Supplementary Figure S1). After that, the EMT-related proteins (fibronectin, E-cadherin, vimentin) were measured and we found that the level of fibronectin and vimentin were up-regulated by TGF-β1 in BEAS-2B (*P* < 0.0001, *P* < 0.0001) and 16HBE (*P* < 0.0001, *P* < 0.0001) cells, while these up-regulation were sharply inhibited by miR-203a-3p re-expression (*P* < 0.0001, *P* < 0.0001 in BEAS-2B cells and *P* < 0.0001, *P* < 0.0001 in 16HBE cells). Besides that, E-cadherin protein was inhibited by TGF-β1 in BEAS-2B (*P* = 0.0017) and 16HBE (*P* = 0.0002) cells, but was increased by following miR-203a-3p mimics transfection (*P* = 0.0218, *P* = 0.0195; Figure 3B). Therefore, we implied that miR-203a-3p blocked TGF-β1-induced EMT in bronchial epithelial cells.

### SIX1 was a target of miR-203a-3p

**Figure 4:**
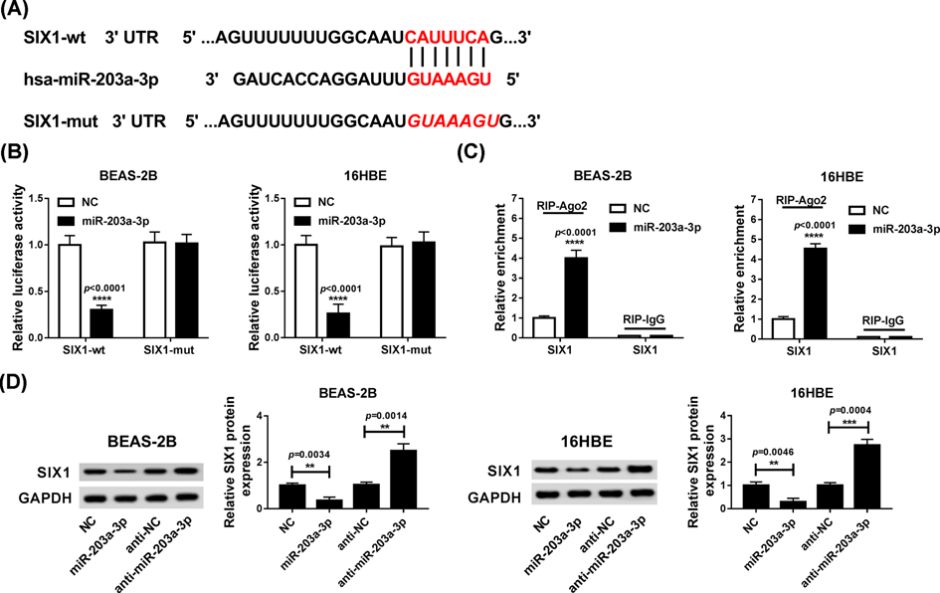
SIX1 was directly targeted by miR-203a-3p (**A**) The putative binding sites between miR-203a-3p and SIX1 wt 3′UTR, and the mutant type of SIX1 3′UTR were exhibited. (**B**) Luciferase activity was measured in BEAS-2B and 16HBE cells co-transfected with SIX1 wt or SIX1 mut and NC or miR-203a-3p. (**C**) RIP assay was performed to detect the enrichment of SIX1 in BEAS-2B and 16HBE cells co-transfected with NC and miR-203a-3p. (**D**) The expression of SIX1 was examined in BEAS-2B and 16HBE cells transfected with NC, miR-203a-3p, anti-NC or anti-miR-203a-3p by Western blot. **P < 0.01, ***P < 0.001, ****P < 0.0001.

Based on the negative correlation between miR-203a-3p and SIX1, the relationship between them was investigated. Through using the TargetScan Prediction tool, the binding sites between miR-203a-3p and SIX1 were predicted (Figure 4A). To further validate this prediction, the dual-luciferase reporter assay was conducted and results indicated that co-transfection of miR-203a-3p and SIX1-wt dramatically decreased the luciferase activity of BEAS-2B (*P* < 0.0001) and 16HBE (*P* < 0.0001) cells. However, there was no significant changes of luciferase activity in SIX1-mut group (Figure 4B). Moreover, RIP assay revealed that overexpression of miR-203a-3p resulted in marked increase in SIX1 enrichment after Ago2 RIP (*P* < 0.0001, *P* < 0.0001), whereas its efficacy was lost in response to IgG RIP (Figure 4C). Furthermore, the level of SIX1 protein was found to be increased by miR-203a-3p inhibition (*P* = 0.0014, *P* = 0.0004), but was decreased by miR-203a-3p overexpression in BEAS-2B (*P* = 0.0034) and 16HBE (*P* = 0.0046) cells (Figure 4D). Taken together, miR-203a-3p directly targeted SIX1 and down-regulated SIX1 level.

### SIX1 knockdown blocked TGF-β1-induced EMT in BEAS-2B and 16HBE cells

**Figure 5:**
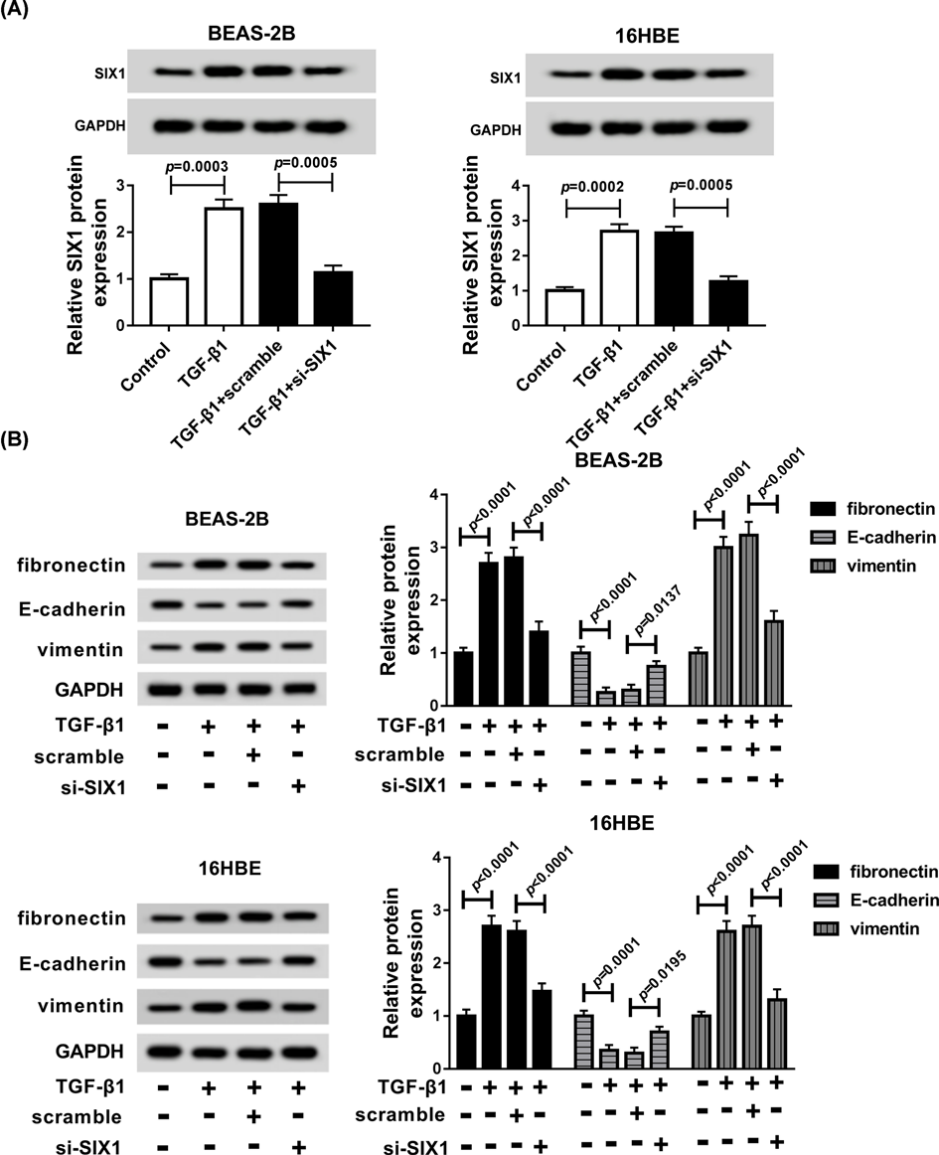
SIX1 repression inhibited TGF-β1-induced EMT in BEAS-2B and 16HBE cells BEAS-2B and 16HBE cells were treated with TGF-β1, TGF-β1 + scramble, or TGF-β1 + si-SIX1. (**A**) The level of SIX1 protein was determined by Western blot in BEAS-2B and 16HBE cells. (**B**) The expression of fibronectin, E-cadherin and vimentin were measured in BEAS-2B and 16HBE cells using Western blot.

To clarify the function of SIX1 on EMT in asthma, BEAS-2B and 16HBE cells were transfected with si-SIX1 before TGF-β1 treatment, and we observed that SIX1 level was up-regulated by TGF-β1 (*P* = 0.0003, *P* = 0.0002), but was down-regulated by following SIX1 deletion in BEAS-2B (*P* = 0.0005) and 16HBE (*P* = 0.0005) cells (Figure 5A). Besides, SIX1 was also decreased in BEAS-2B and 16HBE cells transfected with si-SIX1 (*P* = 0.0003; Supplementary Figure S1). Afterward, Western blot analysis showed SIX1 deletion markedly reversed TGF-β1-induced up-regulation of fibronectin and vimentin protein (*P* < 0.0001, *P* < 0.0001 in BEAS-2B cells and *P* < 0.0001, *P* < 0.0001 in 16HBE cells), down-regulation of E-cadherin protein in BEAS-2B (*P* = 0.0137) and 16HBE (*P* = 0.0195) cells (Figure 5B). These results demonstrated that SIX1 knockdown impeded EMT in TGF-β1-induced BEAS-2B and 16HBE cells.

### SIX1 overexpression abated miR-203a-3p overexpression-mediated inhibition of EMT in TGF-β1-induced BEAS-2B and 16HBE cells

**Figure 6:**
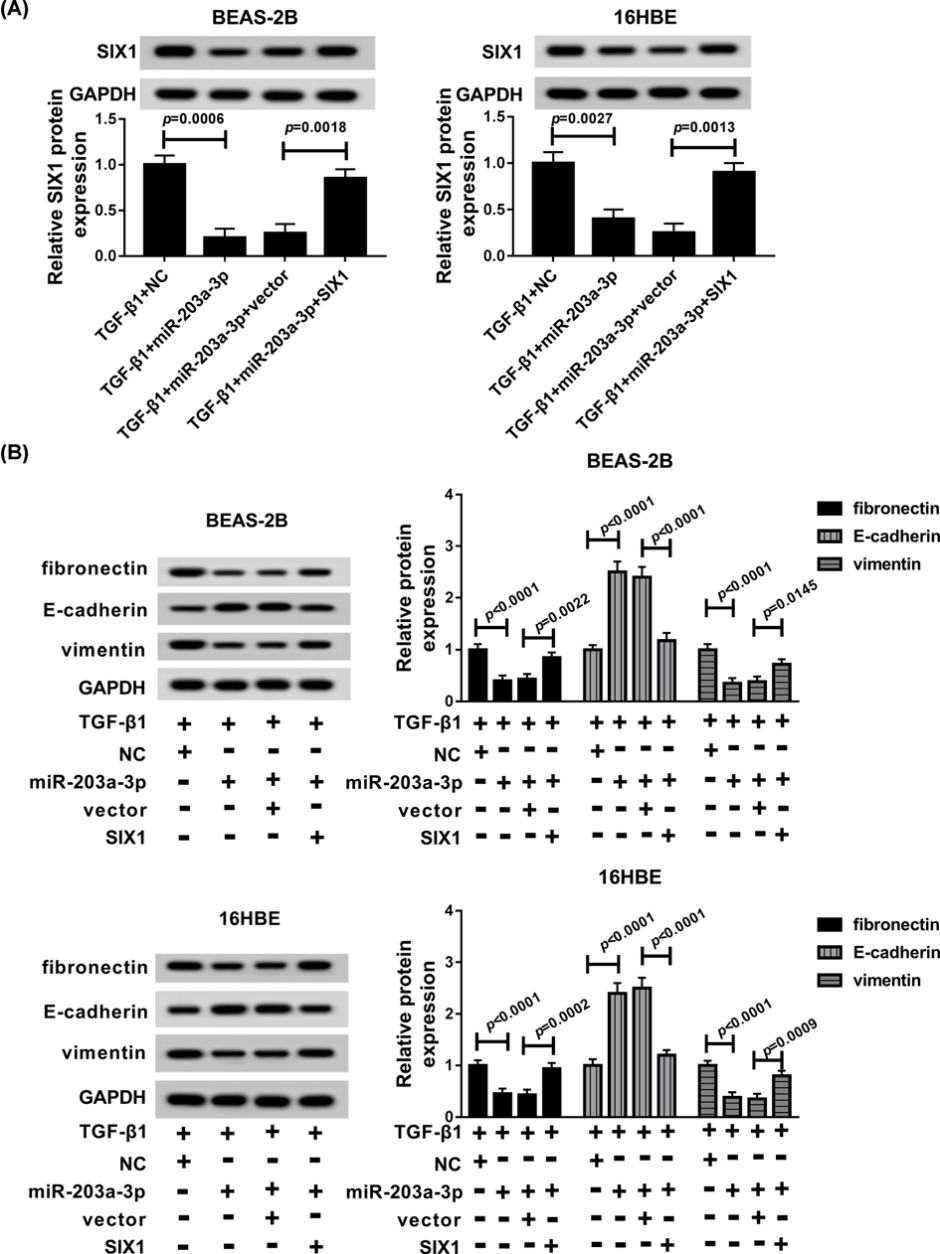
SIX1 up-regulation abrogated the effect on EMT by miR-203a-3p overexpression (**A** and **B**) Western blot assay was conducted to detect the expression of SIX1 and EMT-related proteins (fibronectin, E-cadherin, vimentin) in TGF-β1-induced BEAS-2B and 16HBE cells transfected with NC, miR-203a-3p, miR-203a-3p + vector or miR-203a-3p + SIX1.

Based on the axis of miR-203a-3p/SIX1 in BEAS-2B and 16HBE cells, we further investigated whether SIX1 involved in miR-203a-3p mediated regulation on the EMT in asthma. First, NC, miR-203a-3p, miR-203a-3p + vector or miR-203a-3p + SIX1 were transfected into the TGF-β1-induced BEAS-2B and 16HBE cells, respectively. Next, Western blot analysis revealed that the level of SIX1 was prominently suppressed by miR-203a-3p up-regulation (*P* = 0.0006, *P* = 0.0027), which was reversely promoted by the introduction of SIX1 in BEAS-2B (*P* = 0.0018) and 16HBE (*P* = 0.0013) cells treated with TGF-β1 (Figure 6A). Immediately, fibronectin, E-cadherin and vimentin levels were detected, and results disclosed that miR-203a-3p repressed the expression of fibronectin (*P* < 0.0001, *P* < 0.0001) and vimentin (*P* < 0.0001, *P* < 0.0001) and enhanced the level of E-cadherin (*P* < 0.0001, *P* < 0.0001) in TGF-β1-induced BEAS-2B and 16HBE cells, while miR-203a-3p-mediated effects on EMT-related expression were reversed by the transfection of SIX1 (Figure 6B). In conclusion, SIX1 could restore the effect on EMT triggered by miR-203a-3p up-regulation *in vitro*.

### MiR-203a-3p/SIX1 axis regulated EMT process in TGF-β1-induced bronchial epithelial cells through activating Smad3 pathway

**Figure 7:**
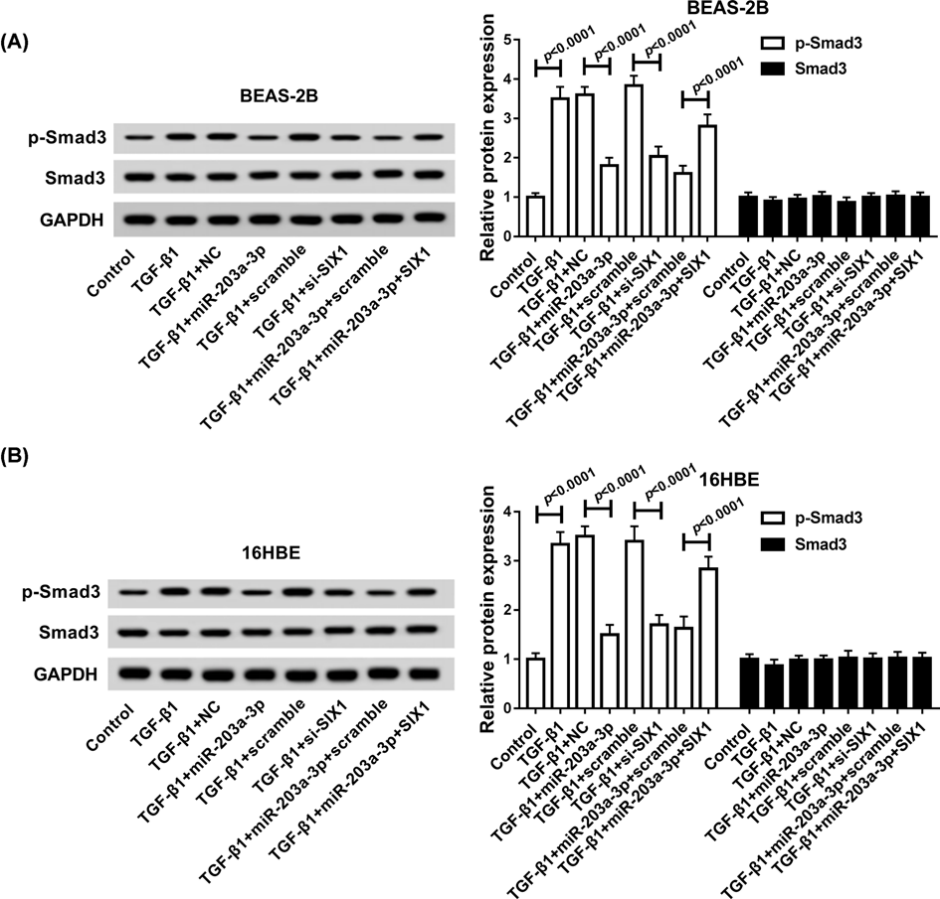
MiR-203a-3p/SIX1 axis regulated TGF-β1-induced EMT via Smad3 pathway (**A** and **B**) Western blot assay was carried out to detect the levels of Smad3 and p-Smad3 in BEAS-2B and 16HBE cells transfected with TGF-β1, TGF-β1 + NC, TGF-β1 + miR-203a-3p, TGF-β1 + scramble, TGF-β1 + si-SIX1, TGF-β1 + miR-203a-3p + scramble or TGF-β1 + miR-203a-3p + SIX1.

To further elucidate the underlying molecular mechanism of miR-203a-3p/SIX1 axis on EMT in TGF-β1-treated bronchial epithelial cells, the expression of proteins involved in Smad3 pathway (Smad3 and p-Smad3) were detected by Western blot assay. Then, results suggested the increase in p-Smad3 protein induced by TGF-β1 treatment (*P* < 0.0001, *P* < 0.0001) could be reversed by miR-203a-3p re-expression (*P* < 0.0001, *P* < 0.0001) or SIX1 deletion (*P* < 0.0001, *P* < 0.0001) in BEAS-2B and 16HBE cells. Meanwhile, we also observed that SIX1 overexpression could attenuate miR-203a-3p restoration-mediated reduction of p-Smad3 expression in TGF-β1 treated BEAS-2B (*P* < 0.0001) and 16HBE (*P* < 0.0001) cells, while no changes of Smad3 expression were observed in each group (Figure 7A,B). These data proved that miR-230a-3p/SIX1 axis regulated EMT in TGF-β1-induced bronchial epithelial cells by Smad3 pathway.

## Discussion

Asthma is a common chronic respiratory disease, which is characterized by varying degrees of chronic airway inflammation and airway remodeling. TGF-β1 is a well-recognized important regulator of airway remodeling in asthma, which can directly controlling the deposition of collagen in the airway wall and inducing the fibrosis formation [23]. Besides that, the involvement of TGF-β1/Smad3 signaling pathway in the formation of asthmatic airway remodeling has been identified [24]. Therefore, TGF-β1 is an important factor for the development of asthma. EMT was suggested as a pathogenesis of asthma, which could be induced by chronic airway inflammation and participate in airway remodeling [25,26]. Previous studies revealed that TGF-β1 involved in airway remodeling through inducing EMT in HBE cells [27,28]. In our study, we found that TGF-β1 elevated fibronectin and vimentin levels and reduced E-cadherin level in BEAS-2B and 16HBE cells, suggesting that TGF-β1 enhanced EMT in HBE cells, which was consistent with the previous studies [27,28].

TGF-β1-induced EMT can be modulated by many molecules, including miRNAs [20,21]. Until now, the role of many miRNAs in the development of asthma has been investigated. For example, miR-145-5p exacerbated asthma pathogenesis through suppressing KIF3A expression in mouse airway epithelial cells [29]. MiR-19 mediated HMGB1-induced proliferation and migration of human airway smooth muscle cells to affect the airway remodeling in asthma by targeting PTEN [30]. In the present study, miR-203a-3p was down-regulated in asthma serums. Besides, TGF-β1 treatment induced the reduction of miR-203a-3p in HBE cells. MiR-203a-3p has been found to be associated with several lung diseases. For instance, miR-203a-3p inhibited cell proliferation, migration and invasion in lung adenocarcinoma by FBXL19-AS1/miR-203a-3p axis [31]. MiR-203a-3p served as a target of LINC00342 to inhibit cell proliferation, colony formation, migration and invasion in non-small cell lung cancer [32]. Recently, bioinformatics analysis indicated that the expression of miR-203a-3p was dramatically decreased in the bronchial epithelial cells [14]. Therefore, it is imperative to disclose the role of miR-203a-3p in asthma. Our functional experiments showed the promotion of EMT induced by TGF-β1 treatment could be reversed by miR-203a-3p re-expression HBE cells, implying that miR-203a suppressed EMT process in HBE cells.

Interestingly, we also found that SIX1 level was opposite correlated with miR-203a-3p expression in asthma serum samples. Furthermore, SIX1 was confirmed to be a target of miR-203a-3p. SIX1 was reported to inhibit tumor invasion and might be a novel therapeutic target in pancreatic cancer [33]. Also, previous study indicated that SIX1 participated in TGF-β1-induced EMT in lung cancer tissues [20]. SIX1 level was up-regulated in asthma serum samples and induced by TGF-β1 treatment in human bronchial epithelial cells. Besides, SIX1 knockdown could block TGF-β1-induced EMT. These data exhibited that SIX1 played an essential role in TGF-β1-induced EMT and could be a potential target of treatment for asthma. More than that, restoration experiment proved that the effect on EMT induced by miR-203a-3p up-regulation could be rescued by SIX1 overexpression.

As reported, Smad3 was an important regulator of TGF-β1 signaling pathway [34] and could be activated by TGF-β1 to regulate various cellular functions [35]. Moreover, the phosphorylation of the C-terminal of Smad3 was the main action of TGF-β signaling pathway [36,37]. In the present study, we found TGF-β1 promoted the level of p-Smad3 protein, while the increase of p-Smad3 protein induced by TGF-β1 treatment could be reversed by miR-203a-3p re-expression or SIX1 deletion in human bronchial epithelial cells. Meanwhile, SIX1 overexpression could attenuate miR-203a-3p restoration-mediated reduction of p-Smad3 expression in TGF-β1 treated BEAS-2B and 16HBE cells, while no changes of Smad3 expression were observed in each group. These findings proved that TGF-β1 regulated Smad3 pathway via miR-203a-3p/SIX1 axis.

## Conclusions

Our findings demonstrated that miR-203a-3p was down-regulated in asthma serums, and overexpressed miR-203a-3p inhibited TGF-β1-induced EMT in asthma by regulating Smad3 pathway through targeting SIX1, suggesting a promising therapeutic approach for bronchial epithelial cell EMT in asthma. However, all experiments in the present study were conducted *in vitro*, there was no *in vivo* data to support these results. Hence, the further research in asthma patients or animal models needs to be performed in future.

## Supplementary Material

Supplementary Figure S1

## Abbreviations

EMT: epithelial–mesenchymal transition
RISC: RNA-induced silencing complex
SIX1: Sine oculis homeobox homolog
TGF-β1: transforming growth factor-β
