# Expression of Concern: MiR-203a-3p regulates TGF-β1-induced epithelial-mesenchymal transition (EMT) in asthma by regulating Smad3 pathway through SIX1

**DOI:** 10.1042/BSR-2019-2645_EOC

**Published:** 2024-11-06

**Keywords:** asthma, EMT, miR-203a-3p, SIX1, Smad3, TGF-β1

The Editorial Office has been made aware of potential issues surrounding the scientific validity of this paper “MiR-203a-3p regulates TGF-β1-induced epithelial–mesenchymal transition (EMT) in asthma by regulating Smad3 pathway through SIX1” DOI: 10.1042/BSR20192645, hence has issued an expression of concern to notify readers whilst the Editorial Office investigates. It has been noted that the Western blots appear to have minimal background detail, and resemble those in other publications by different authors. The authors were contacted regarding the concerns, however so far the Editorial Office has not received a response.

