# Supplementary material for: MiR-203a-3p regulates TGF-β1-induced epithelial–mesenchymal transition (EMT) in asthma by regulating Smad3 pathway through SIX1
Source: Biosci Rep. 2020 Feb 28;40(2):BSR20192645. doi: 10.1042/BSR20192645 (PMC7048677; doi:10.1042/BSR20192645)
Supplement: Supplementary Figure S1 [file BSR-2019-2645_supp.pdf]

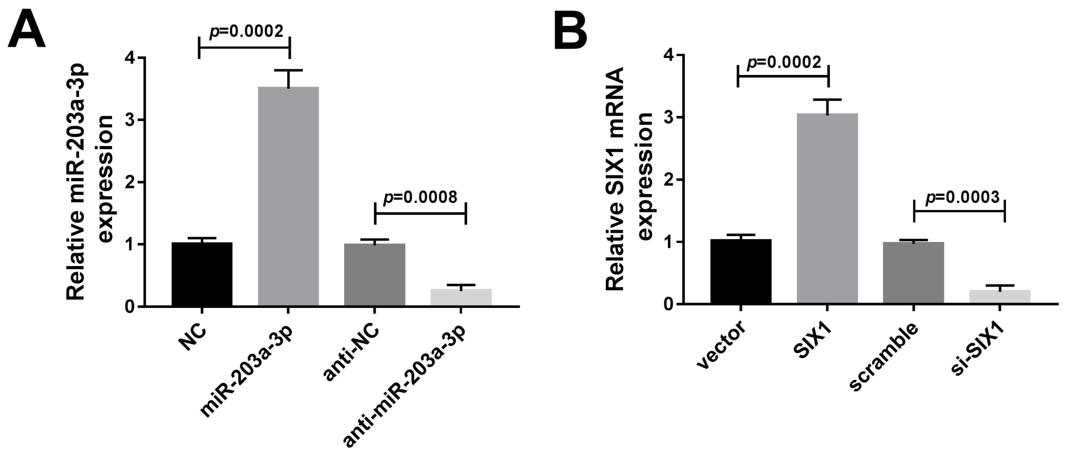
A
p=0.0002
p=0.0008
Relative miR-203a-3p expression
4
3
2
1
0
NC
miR-203a-3p
anti-NC
anti-miR-203a-3p
B
p=0.0002
p=0.0003
Relative SIX1 mRNA expression
4
3
2
1
0
vector
SIX1
scramble
si-SIX1

Figure S1. The transfection efficiency of miR-203a-3p and SIX1. (A) The level of miR-203a-3p was detected using qRT-PCR in 16HBE cells transfected with NC, miR-203a-3p, anti-NC, or anti-miR-203a-3p. (B) The expression of SIX1 was detected using qRT-PCR in 16HBE cells transfected with vector, SIX1, scramble, or si-SIX1.
